# The effect of lidocaine intraoperative infusion on quality of postoperative sleep in patients undergoing thyroidectomy: a randomized controlled trial

**DOI:** 10.1186/s12871-023-02109-w

**Published:** 2023-05-09

**Authors:** Caiqun Shao, Longxiang Lin, Mengmeng Chen, Ning Wang, Wangning Shangguan

**Affiliations:** Department of Anesthesiology and Perioperative Medicine, The Second Affiliated Hospital, Yuying Children’s Hospital of Wenzhou Medical University, 109 West Xueyuan Road, Wenzhou, 325027 China

**Keywords:** Lidocaine, Thyroidectomy, Sleep quality, Postoperative complications

## Abstract

**Background:**

The incidence of thyroid nodules has increased significantly in recent years, and surgical removal is a common treatment. Postoperative sleep disturbance is still a serious problem in the current surgical environment. In this study, we explored whether intraoperative lidocaine infusion could improve the quality of sleep over 7 days and 30 days after surgery and postoperative recovery for patients undergoing thyroid surgery.

**Methods:**

Seventy patients who underwent thyroid surgery from October 2020 to June 2021 were randomly assigned to the lidocaine or the normal saline group, 35 cases in each group. Patients enrolled in this study were randomized to receive either system lidocaine (a bolus of 1.5 mg·kg^− 1^, followed by an infusion of 2 mg·kg^− 1^·h^− 1^ until the end of the surgical procedure) or identical volumes and rates of normal saline. The primary endpoint was the Pittsburgh Sleep Quality Index (PSQI) scores. Secondary endpoints included intraoperative remifentanil consumption, whether there was a cough within 5 min after extubation and the cough scores, postoperative pain scores, the incidence of postoperative nausea and vomiting (PONV).

**Results:**

Totally seventy cases were enrolled and eventually sixty-eight cases were analyzed. PSQI scores did not change significantly over time (F = 2.799, *P* = 0.069); also, there was no significant difference in PSQI scores between two groups in the entire 30 days follow-up period (F = 0.174, *P* = 0.678). Further, there was no interaction between the time points and the intervention (F = 0.649, *P* = 0.513). Similarly, intraoperative remifentanil consumption, the incidence of cough and postoperative pain scores, were comparable between the two groups (all *P* > 0.05); while patients in the lidocaine group showed significantly lower cough scores (*P* = 0.042) and lower incidence of PONV (*P* = 0.015).

**Conclusions:**

Systemic lidocaine infusion might not improve the sleep quality and reduce postoperative pain over 7 days or 30 days after the operation of patients who underwent thyroid surgery, but it can reduce postoperative complications and improve the quality of recovery. Furthermore, sleep quality of patients wasn’t impaired significantly in the entire 30 days follow-up period after thyroid surgery compared with baseline values.

**Trial registration:**

Registered in the Chinese Clinical Trial Registry (http://www.chictr.org.cn, identifier: ChiCTR2000039764, date: 08/11/2020)

**Supplementary Information:**

The online version contains supplementary material available at 10.1186/s12871-023-02109-w.

## Background

In recent years, the incidence of thyroid nodules has increased substantially, with an incidence of about 4% in adults [1], surgical removal has become a common treatment. Previous studies have demonstrated that intraoperative lidocaine infusion could improve the quality of recovery following thyroidectomy, including attenuating cough and the hemodynamic changes during the extubation period [2], reducing postoperative pain and sensory impairment [3, 4], decreasing the incidence of nausea [4],but the effect on postoperative sleep quality is rarely reported. In fact, study has shown that thyroid disorders may affect the sleep quality [5].

Lidocaine is an amide-based local anesthetic commonly used for local anesthesia, which could also act on the immune system, reducing the production and release of pro-inflammatory cytokines. Prior studies have found that lidocaine can decrease the serum levels of cytokines such as IL-1, IL-6, and TNF in a dose-dependent manner [6–8].

Indeed, there is now evidence that the inflammatory reaction caused by surgical trauma is an important factor for postoperative sleep disturbance [9, 10]. Improvement of sleep quality after IL-6 or TNF receptor antagonists treatments in patients with autoimmune diseases didn’t appear to directly result from decreased disease activity, suggesting that abnormal regulation of IL-6 and TNF may be associated with sleep disturbances [11, 12].

Therefore, our study aimed at evaluating the association between intraoperative lidocaine infusion and the sleep quality of patients following thyroid surgery. We hypothesized that intraoperative lidocaine infusion could improve the quality of sleep and postoperative recovery for patients undergoing thyroid surgery.

## Materials and methods

### Ethics approval and consent to participate

This study was approved by the Ethics Committee of The Second Affiliated Hospital and Yuying Children’s Hospital of Wenzhou Medical University (Ethics No. LCKY2020-234). All methods were carried out in accordance with the Declaration of Helsinki. All patients were informed about the study and signed the informed consents. This study was registered on Chinese Clinical Trial Registry (http://www.chictr.org.cn, identifier: ChiCTR2000039764, date: 08/11/2020), following the CONSORT guidelines [13].

### Participants

After written informed consent was obtained, patients undergoing scheduled thyroid surgery from October 2020 to June 2021 at the Second Affiliated Hospital and Yuying Children’s Hospital of Wenzhou Medical University were enrolled. The inclusion criteria were as follows: (1) aged 18–45 years old; (2) female; (3) with the BMI between 18.5 and 23.9 kg/m^2^; (4) received elective thyroid surgery; (5) with American Society of Anesthesiology (ASA) physical status classification grade I–II; (6) signed informed consent. The exclusion criteria included the following: (1) history of allergies to local anesthetics; (2) hyperthyroidism or hypothyroidism; (3) known sleep disorders or pain, use of sedatives, hypnotics or analgesics; (4) congestive heart failure; (5) severe arrhythmia (such as atrial-ventricular block, pre-excitation syndrome, Adam-Stoke syndrome, etc.); (6) hepatic or renal insufficiency.

### Study design

All participants were randomly divided into lidocaine or normal saline group in a 1:1 ratio by the investigator using a computer-generated sequence. The sequential numbers from 1 to 70 for participants were marked outside of each individual envelope. Based on the computer-generated sequence, 35 paper slips marked with #1 (normal saline group) and 35 slips marked with #2 (lidocaine group) were sealed inside of envelopes. On the day of study, a nurse who was not involved in the administration and observation opened an envelope with the smallest sequential number and prepared the medicines based on the number appeared on the slip, either #1 or #2. Then, the medicines were handed over to an anesthesiologist who would administrate it and perform general anesthesia. The normal saline was matched to the lidocaine for color, smell, volume and method of administration. Patients, the anesthesiologist who performed anesthesia and another designated anesthetist who observed and recorded the data were all blinded to the study arms. All operations involved in the study were completed between 08:00 am and 12:00 noon.

After entering into the operating room, all patients were monitored for electrocardiograph (ECG), pulse oximeter (SpO_2_), and noninvasive blood pressure (NBP). After preoxygenation with oxygen 100% for three minutes, anesthesia was induced with sufentanil (0.3 µg·kg^− 1^), propofol (2.5 mg·kg^− 1^), cisatracurium (0.2 mg·kg^− 1^) and 1.5 mg·kg^− 1^ lidocaine (Sinopharm Group Rongsheng Pharmaceutical Co., Ltd., Henan Wuzhi, batch number: H20043676) over 10s (lidocaine group) or the same volume of normal saline (normal saline group), followed by 2 mg·kg^− 1^·h^− 1^ continuous lidocaine (lidocaine group) or normal saline (normal saline group) until the end of the surgery. All patients were monitored for the adverse events associated with lidocaine administration during the study.

The patients were then intubated, and general anesthesia was maintained with 1.5–2% sevoflurane in an oxygen/air mixture and 0.1–2 µg·kg^− 1^·min^− 1^ remifentanil. After intubation, tidal volume was adjusted to 6–8 ml·kg^− 1^, and the ventilator rate was adjusted to maintain the end-tidal CO_2_ at 35–45 mmHg. At the beginning of surgery, all patients in the study received 0.5 mg·kg^− 1^ ketorolac tromethamine for postoperative analgesia. Neuromuscular blocking effect was reversed with atropine (0.5 mg) and neostigmine (1 mg) before extubation. The endotracheal tube was removed when the patient regained consciousness and was able to breathe spontaneously after the operation. After extubation, all patients were transferred to the postoperative anaesthesia care unit (PACU).

### Primary and secondary endpoints

The primary endpoint of this study was the sleep quality. Sleep quality was assessed using the Pittsburgh Sleep Quality Index (PSQI) questionnaire (Supplementary Material 1 and 2) [14, 15]). Face‑to‑face survey was conducted the day before the surgery. At the time of the follow-up visit ( on the 7th and 30th day after surgery), all patients were asked to assess their sleep quality via telephone by the same designated anesthetist who was blinded to the assigned groups. With higher degree of internal homogeneity, overall consistency and clinical validity than any other test available, the PSQI, originally described by Buysse in 1989, has established itself as the main tool for the assessment of quality of sleep [14, 15]. There are 19 questions belonging to 7 sub-categories. Each sub-category is rated on a scale of 0 to 3, and the total score for the sub-categories is 21 points. Higher scores indicate a lower quality of sleep. The 7 sub-categories of the PSQI are as follows: subjective sleep quality, sleep latency, sleep duration, habitual sleep efficiency, sleep disturbance, sleep medication use, and daytime dysfunction.

Secondary endpoints included intraoperative remifentanil consumption, whether there was a cough within 5 min after extubation and the cough scores, postoperative pain scores and the incidence of postoperative nausea and vomiting (PONV).

A 10 cm visual analogue scale was used to evaluate postsurgical pain at the surgical site in the 7th and 30th day after surgery [16]. Cough results were sorted by the modified four point Minogue scale [17]: grade 1 indicates no cough; grade 2 (mild) is coughing once or twice; grade 3 (moderate) is fewer than four non-sustained coughs lasting 1–2 s each, or overall coughing lasting less than 5s; and grade 4 (severe) is at least four coughs lasting at least 2s, or overall coughing duration being more than 5s.

### Sample size

Our preliminary study showed that the standard deviation of the sample was 4.03 scores. The PSQI has a minimum clinically important difference (MCID) of ≥ 3 [18], so we chose 3 scores as the estimated variability between the two groups. According to the calculation of the sample size (n_1_ = n_2_ = 2*[(u_α_+u_β_)*(*σ*/*δ*)]^2^+1/4*u_α_^2^), 30 patients for each group were required, assuming a two-sided Type I error (α) of 0.05 and a power of 80%. The potential loss was expected during follow-up or due to drop out; therefore, a total of 70 patients were enrolled in this study.

### Statistical analysis

SPSS 23.0 (IBM Corp, Armonk, NY, United States) and GraphPad Prism 9.0 statistical software were used for data processing and statistical analysis. Patients’ characteristics, types of thyroidectomy, pathology result, intraoperative and postoperative variables were summarized through descriptive statistics. In addition, t–tests or Manne Whitney U tests were used to compare continuous variables between two groups. Fisher**’**s exact text or χ^2^ test was used to evaluate the associations between categorical variables. Categorical data were expressed as n (%) and analyzed with the χ^2^ test; continuous data were expressed as mean values ± standard deviation (SD), and two independent samples were analyzed with the t-test. Linear mixed model was used to evaluate the PSQI scores differences between two groups and changes after surgery. *P* < 0.05 was considered statistically significant.

## Results

**Fig. 1:**
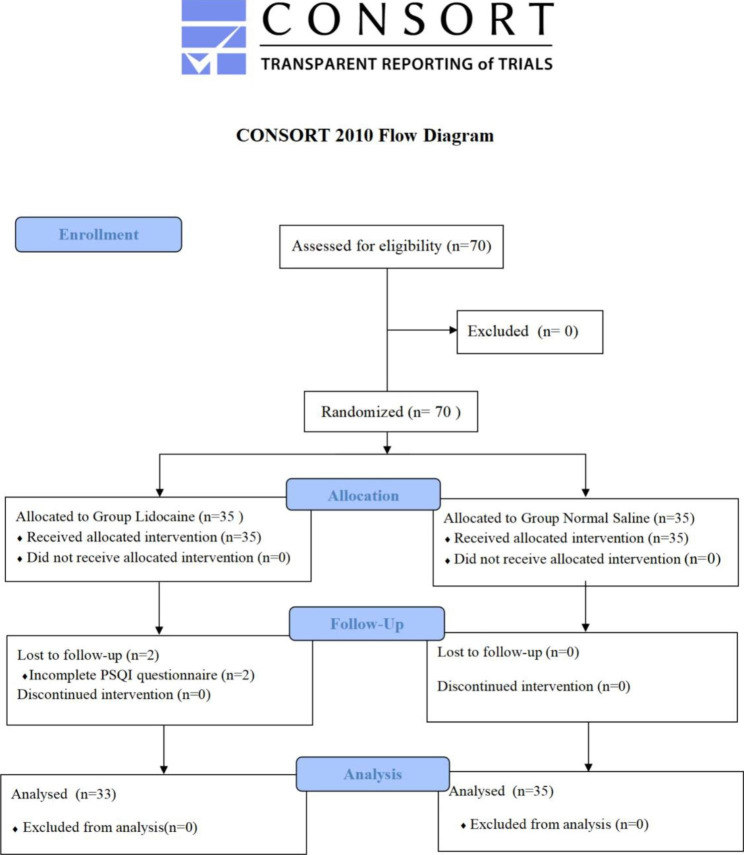
Consolidated Standards of Reporting Trials (CONSORT) flowchart describing patients progress through the study

Totally 70 cases were enrolled from October 2020 to June 2021 and all participated in the PSQI questionnaire before surgery, two patients in the lidocaine group were lost in the entire 30 days follow-up period after surgery, eventually sixty-eight cases were analyzed (Fig. 1). Patients’ characteristics, including age, body mass index (BMI), ASA physical status, preoperative thyroid function test results, intraoperative variables, were similar between two groups (Table 1). Types of thyroidectomy, postoperation pathological results and cervical lymph node metastasis were also similar between two groups (Table 2).

**Table 1:** Patients’ characteristics for both groups

		Normal saline group (n = 35)	Lidocaine group (n = 33)	*P*
Age (year)		39 (33–42)	36 (32–42)	0.190
BMI (kg/m^2^)		21 (19.5–22.9)	21 (19.9–21.9)	0.912
Duration of operation (min)		40 (34–47)	40 (32.5–45.0)	0.685
Duration of anesthesia (min)		77 (66–86)	76 (68.5–86.0)	0.868
ASA physical status (n, %)	I	34 (97.1)	30 (90.9)	0.278
	II	1 (2.9)	3 (9.1)	
TT_3_ (ng/ml)		1.09 ± 0.20	1.01 ± 0.14	0.075
TT_4_ (µg/dl)		8.46 ± 1.55	8.11 ± 1.66	0.368
FT_3_ (pg/ml)		3.21 ± 0.28	3.15 ± 0.26	0.421
FT_4_ (ng/dl)		1.21 ± 0.16	1.24 ± 0.18	0.668
TSH (µ IU/ml)		1.30 (0.88–2.31)	1.28 (0.80–2.13)	0.727

**Table 2:** Types of thyroidectomy and postoperation pathological report of patients in two groups

		Normal saline group (n = 35)	Lidocaine group (n = 33)	*P*
Types of thyroidectomy (n, %)	nodule resection	0	1 (3.0)	0.739
	Subtotal thyroidectomy	34 (97.1)	31 (94.0)	
	Total thyroidectomy	1 (2.90)	1 (3.0)	
Pathological results (n, %)	Benign tumor	11 (31.4)	11 (33.3)	0.868
	Malignancy	24 (68.6)	22 (66.7)	
lymph node metastasis (n, %)	Yes	6 (17.1)	7 (21.2)	0.670
	No	29 (82.9)	26 (78.8)	

Data are presented as number (percentage)

ASA American Society of Anesthesiologists physical status, BMI Body mass index, TT3 Total triiodothyronine, TT4 Total thyroxine, FT3 Free triiodothyronine, FT4 Free thyroxine, TSH Thyroid-stimulating hormone, SD standard deviation. Reference ranges, TT3(0.60–1.81ng/mL), TT4(4.50–10.90 µg/dl), FT3 (2.30–4.20 pg/mL), FT4 (0.89–1.76 ng/dL), TSH (0.550–4.780 µ IU/mL), were used. Data are presented as median (interquartile range), number (percentage) or mean ± SD

**Fig. 2:**
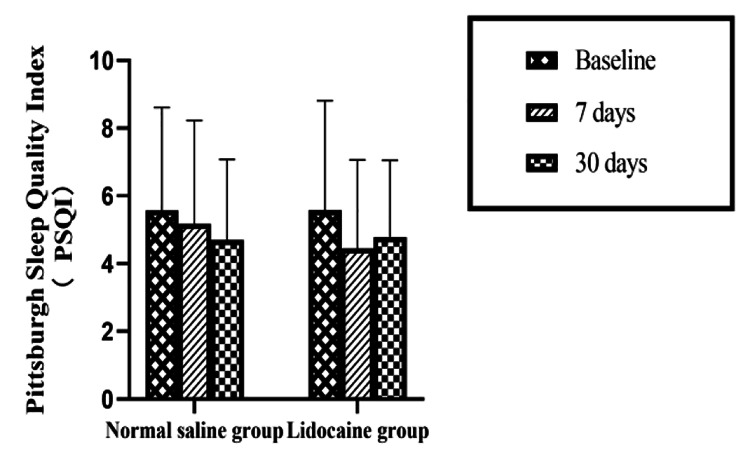
PSQI scores at three time points in the two groups, values are expressed as mean ± SD.

PSQI scores didn’t change significantly over time (F = 2.799, *P* = 0.069); also, there was no significant difference in PSQI scores between two groups in the entire 30 days follow-up period (F = 0.1, *P* = 0.678). Further, there was no interaction between the time points and the intervention (F = 0.649, *P* = 0.513). Compared with the preoperative point, the PSQI scores of patients in two groups were lower on the 7th and 30th day after surgery (5.57 ± 3.04 vs. 5.18 ± 3.05, 4.70 ± 2.38 for normal saline group; 5.58 ± 3.23 vs. 4.45 ± 2.61, 4.78 ± 2.27 for lidocaine group) (Fig. 2), but there was no statistical difference between two groups. No additional analgesic was used in the two groups in the entire 30 days follow-up period. There was no significant difference in VAS scores between two groups in the 7th and 30th day postoperatively (Table 3).

**Table 3:** The VAS scores in both groups in the 7th and 30th day after surgery

	Normal saline group (n = 35)	Lidocaine group (n = 33)	t	*P*
The VAS scores in the 7th day	1.20 ± 1.45	1.02 ± 1.14	0.603	0.549
The VAS scores in the 30th day	0.47 ± 0.75	0.55 ± 0.84	-0.401	0.690

VAS visual analogue scale, SD standard deviation

Values are expressed as mean ± SD

**Fig. 3:**
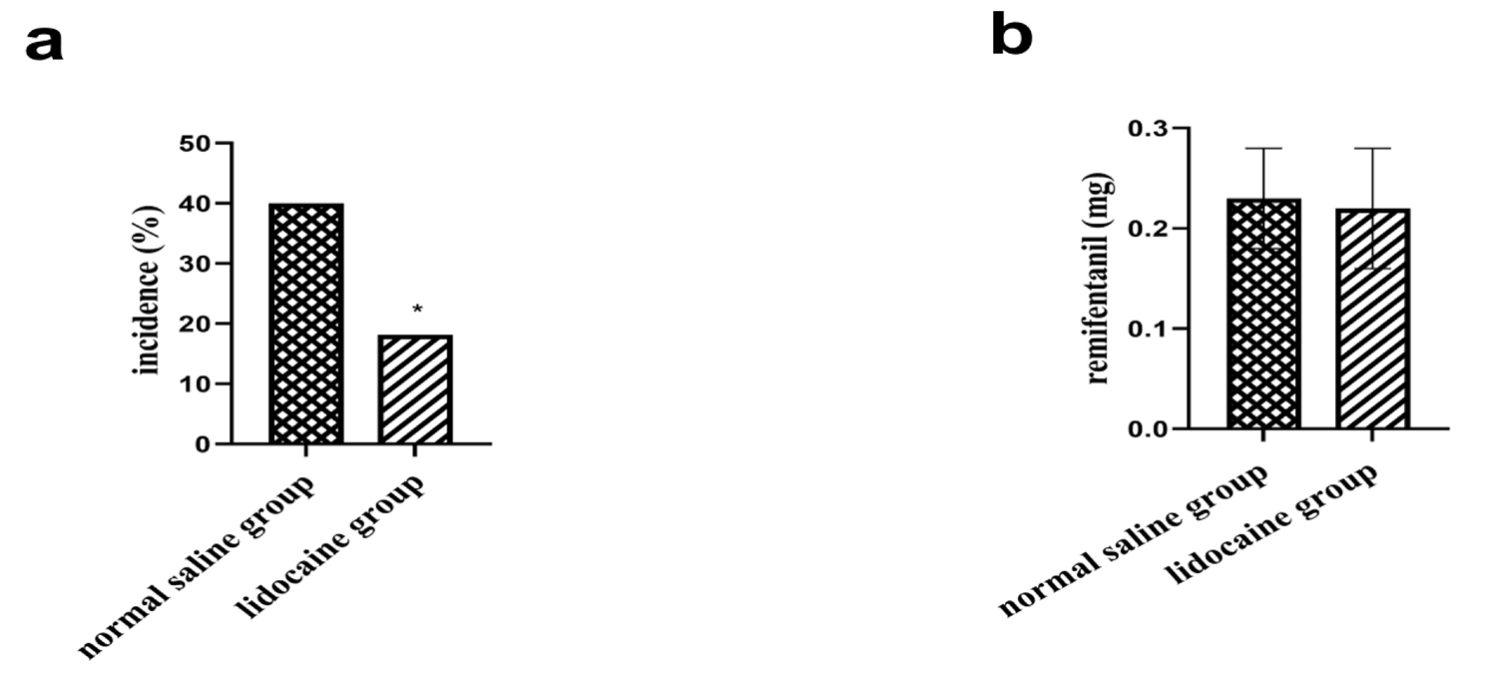
(**a**) The incidence of postoperative nausea and vomiting (PONV) between groups. (**b**) Intraoperative remifentanil consumption between groups. *P < 0.05

The incidence of PONV within 24 h after surgery was 18.18% in the lidocaine group and 40% in the normal saline group(*P* = 0.048; Fig. 3a). In terms of the intraoperative remifentanil consumption, the mean ± SD in the lidocaine group was 0.22 ± 0.06 mg, whereas in the normal saline group, the mean ± SD was 0.23 ± 0.05 mg (*P* = 0.269; Fig. 3b).

There was no significant difference in the incidence of cough within 5 min after extubation between groups (Table 4). But the cough scores were lower in the lidocaine group than that in the normal saline group (*P* = 0.042) (Table 5).

**Table 4:** The incidence of cough within 5 min after extubation between groups

	Cough		Incidence(%)	χ^2^	*P*
	No	Yes			
Normal saline group (n = 35)	24	11	31.43	3.683	0.055
Lidocaine group (n = 33)	29	4	12.12		

Data are presented as counts (n)

**Table 5:** The cough scores between groups

	Cough scores				Average ranks	Z	*P*
	1	2	3	4			
Normal saline group (n = 35)	24	7	4	0	37.91	-2.029	0.042
Lidocaine group (n = 33)	29	4	0	0	30.88		

Data are presented as counts (n)

## Discussion

The current study neither observed significantly impaired sleep quality of patients following thyroid surgery nor demonstrated the effect of intraoperative lidocaine infusion on postoperative sleep quality in patients undergoing thyroidectomy, but it was associated with a significant reduction in the severity of cough within 5 min after extubation, and the incidence of PONV.

Postoperative sleep disturbance remains a challenging problem in surgical procedures. Wang et al. [19] investigated the self-report sleep quality in 107 patients undergoing total joint arthroplasty (TJA). The authors observed significantly impaired sleep quality in TJA patients one week after surgery. Lei**’**s [20] study also showed significantly diminished sleep quality one week after sedative diagnostic upper gastrointestinal endoscopy (UGE). However, the quality of sleep returned to the preoperative baseline values one month after TJA, so did the sedative diagnostic UGE. These findings suggested that transient sleep disturbance was common in the early postoperative period, with subsequent improvement by one-month follow-up after surgery. Therefore, we used the PSQI questionnaire to evaluate the sleep quality of patients undergoing thyroidectomy, before, on the 7th and 30th day after surgery. Generally, the reporting period of PSQI is one month, but the PSQI questionnaire was applied for short-term evaluation for one week in our study. Broderick et al. [21] once evaluated the accuracy of PSQI across different lengths of reporting periods (3-, 7-and 28-days), and found that there was no significant difference of PSQI scores among 3-, 7- and 28-days reporting period. Besides, many previously studies have investigated the short-term sleep quality by using the PSQI questionnaire [19, 20]. Therefore, PSQI could also be administered with confidence for weeklong reporting periods for between-subject analyses.

However, our study didn’t show significantly impaired sleep quality of patients in the entire 30 days follow-up period compared with baseline values. On the other hand, we didn’t observe the effect of systemic lidocaine infusion on postoperative sleep quality in patients following thyroidectomy.

Previous studies indicated that postoperative sleep disturbance could be related to the magnitude and duration of the operation [22–24]. Laparoscopic surgery [24] was associated with a reduced inflammatory response as compared with similar open surgeries [23]. After laparoscopic cholecystectomy, only a slight change in SWS sleep was seen on the first night after surgery and there was no change in REM sleep. Compared with TJA [19], gastroplasty [22] and laparoscopic cholecystectomy [24], thyroidectomy is a mildly invasive operation that causes a milder stress response. Koo et al. [5] also used the PSQI questionnaire to assess sleep quality in patients undergoing thyroid surgery, before, 1 month, 4 months, 10 month and 5 years after surgery. Consistent with our findings, they didn’t observe statistically significant deterioration in sleep quality after operation.

Lidocaine was injected at least 20 min before surgical excision, and was infused continuously until a few hours after surgery in those studies which have demonstrated the significant anti-inflammatory properties of lidocaine [6–8, 25, 26]. However, in our study, the time from anesthesia induction to surgical incision was less than 10 min, and thyroid surgery was all around 30 min, resulting in a short intraoperative lidocaine infusion. It was possible to assume that longer and higher concentrations of lidocaine might have stronger anti-inflammatory effects than shorter infusions or low levels of lidocaine. For these reasons, we didn’t observe the effect of intraoperative lidocaine infusion on postoperative sleep quality in patients following thyroidectomy.

Previous studies have indicated that systemic lidocaine may reduce cough, pain and nausea after surgery [2, 27], which was associated with improvements in the quality of recovery. Our results were similar to these findings. The mechanism of the inhibitory effect of lidocaine on coughing is not completely understood. The vagus nerves richly innervate the larynx, trachea, extrapulmonary and intrapulmonary bronchi, and parenchymal tissues. Mechanical events associated with secretions and edema that may accompany airways inflammation stimulate the vagus nerves leading to cough. Lidocaine, a non-selective inhibitor of voltage-gated sodium channels, can prevent action potentials initiating and conducting to the central terminals within the central nervous system [28]. Possible mechanisms of the anti-PONV effect of intravenous lidocaine in adult patients may be a gastrointestinal recovery and an opioid-sparing effect [29]. In our trial, compared with the normal saline group, the overall intraoperative dosage of remifentanil was fewer in the lidocaine group, although there was no statistically significant difference. Other mechanisms such as anti-inflammatory action and central anti-hyperalgesic effect have been suggested in adult patients.

There were some limitations associated with this study. Firstly, we didn’t evaluate the precise concentration of lidocaine in the plasma. However, the optimal plasma concentration for lidocaine administered intravenously remains unclear, we used a typical dosage and infusion velocity and relatively short infusion duration compared to other studies. Similar to previous studies, in this study, we did not observe any side effects related to lidocaine administration. Secondary, we didn’t take into consideration possible time- and dose-dependent effects of lidocaine. Thirdly, the patient’s vital signs intraoperatively were not collected, but we monitored them closely at all times. Fourthly, our study demonstrated that intraoperative lidocaine infusion might reduce the severity of cough within 5 min after extubation, as well as the incidence of PONV. However, we didn’t verify the power of analysis on these results, which should be considered with caution. Finally, in this study, we only relied on PSQI questionnaire to evaluate the subjective sleep quality of patients before surgery, 7 days and 30 days after surgery.

## Conclusion

In conclusion, systemic lidocaine infusion might not improve the sleep quality and reduce postoperative pain over 7 days or 30 days after the operation of patients who underwent thyroid surgery, but it can reduce postoperative complications and improve the quality of recovery. Furthermore, sleep quality of patients wasn’t impaired significantly in the entire 30 days follow-up period after thyroid surgery compared with baseline values.

## Electronic supplementary material

Below is the link to the electronic supplementary material.

Supplementary Material 1

Supplementary Material 2

## Data Availability

The datasets generated during and/or analysed during the current study are available from the corresponding author on reasonable request.

## Abbreviations

ASA: American Society of Anesthesiology
BMI: Body mass index
ECG: Electrocardiograph
FT_3_: Free triiodothyronine
FT_4_: Free thyroxine
MCID: Minimum clinically important difference
NBP: Noninvasive blood pressure
NREM: Non-rapid eye movement
PACU: Postoperative anaesthesia care unit
PONV: Postoperative nausea and vomiting
PSQI: Pittsburgh Sleep Quality Index
REM: Rapid eye movement sleep
SpO_2_: Pulse oximeter
SWS: Slow-wave sleep
TJA: Total joint arthroplasty
TSH: Thyroid-stimulating hormone
TT_3_: Total triiodothyronine
TT_4_: Total thyroxine
UGE: Upper gastrointestinal endoscopy.
