# Supplementary material for: The effect of lidocaine intraoperative infusion on quality of postoperative sleep in patients undergoing thyroidectomy: a randomized controlled trial
Source: BMC Anesthesiol. 2023 May 9;23:158. doi: 10.1186/s12871-023-02109-w (PMC10169303; doi:10.1186/s12871-023-02109-w)
Supplement: Supplementary file 1 — Supplementary Material 1 [file 12871_2023_2109_MOESM1_ESM.docx]

Pittsburgh Sleep Quality Index (PSQI)

Instructions: The following questions relate to your sleep habits during the past 7 days only. Your answers should indicate the most accurate reply for the majority of days and nights in the past 7 days. Please answer all questions.

During the past 7 days ,

1: when have you usually gone to bed?

2: how long (in minutes) has it taken you to fall asleep each night?

3: when have you usually gotten up in the morning?

4: how many hours of actual sleep did you get that night?(This may be different from the number of hours you spend in bed)

| 1. During the past 7 days, how often have you had trouble sleeping because you... | Not during the past month(0) | Less then once a week(1) | Once or twice a week(2) | Three or more times a week(3) |
| --- | --- | --- | --- | --- |
|  |  |  |  |  |
| 1. Cannot get to sleep within 30 minutes |  |  |  |  |
| 1. Wake up in the middle of the night or early morning |  |  |  |  |
| 1. Have to get up to use the bathroom |  |  |  |  |
| 1. Cannot breath comfortably |  |  |  |  |
| 1. Cough or snore loudly |  |  |  |  |
| 1. Feel to cold |  |  |  |  |
| 1. Feel to hot |  |  |  |  |
| 1. Have bad dreams |  |  |  |  |
| 1. Have pain |  |  |  |  |
| 1. Other reason(s), please describe, including how often you have had trouble sleeping because of this reason(s) |  |  |  |  |
| 1. During the past 7 days, how would you rate your sleep quality overall? | Very  good (0) | Fairly good (1) | Fairly  bad (2) | Very  bad (3) |
|  |  |  |  |  |
| 1. During the past 7 days, how often have you taken medicine to help you sleep? |  |  |  |  |
| 1. During the past 7 days, how often have you had trouble staying awake while driving, eating meals, or engaging in social activity? |  |  |  |  |
| 1. During the past 7 days, how much of a problem has it been for you to keep up enthusiasm to get things done? |  |  |  |  |

| **Component** | **Item** | **Score** | | | |
| --- | --- | --- | --- | --- | --- |
|  |  | **0** | **1** | **2** | **3** |
| A.subjective sleep quality | #6 score | □Very  good | □Fairly  good | □Farily  good | □Very  bad |
| B.sleep latency | #2 Score + #5a Score | □0 | □1~2 | □3~4 | □5~6 |
| C. sleep duration | #4 Score | □＞7h | □6~7h | □5~6h | □＜5h |
| D.sleep efficiency | (total # of hours asleep)/( total # of hours in bed) | □＞85% | □75~85% | □65~75% | □＜65% |
| E.sleep disturbance | # sum of scores 5b to 5j | □0 | □1~9 | □10~18 | □19~27 |
| F.sleep medication use | #7 Score | □0 | □＜once a week | □Once or twice a week | □≥Three times a week |
| G.daytime dysfunction | #8 score + #9 score | □0 | □1~2 | □3~4 | □5~6 |

**PSQI Score=A+B+C+D+E+F+G**
