# Supplementary material for: The effect of lidocaine intraoperative infusion on quality of postoperative sleep in patients undergoing thyroidectomy: a randomized controlled trial
Source: BMC Anesthesiol. 2023 May 9;23:158. doi: 10.1186/s12871-023-02109-w (PMC10169303; doi:10.1186/s12871-023-02109-w)
Supplement: Supplementary file 2 — Supplementary Material 2 [file 12871_2023_2109_MOESM2_ESM.docx]

Pittsburgh Sleep Quality Index (PSQI)

Instructions: The following questions relate to your sleep habits during the past 30 days only. Your answers should indicate the most accurate reply for the majority of days and nights in the past 30 days. Please answer all questions.

During the past 30 days ,

1: when have you usually gone to bed?

2: how long (in minutes) has it taken you to fall asleep each night?

3: when have you usually gotten up in the morning?

4: how many hours of actual sleep did you get that night?(This may be different from the number of hours you spend in bed)

| 1. During the past 30 days, how often have you had trouble sleeping because you... | Not during the past month(0) | Less then once a week(1) | Once or twice a week(2) | Three or more times a week(3) |
| --- | --- | --- | --- | --- |
|  |  |  |  |  |
| 1. Cannot get to sleep within 30 minutes |  |  |  |  |
| 1. Wake up in the middle of the night or early morning |  |  |  |  |
| 1. Have to get up to use the bathroom |  |  |  |  |
| 1. Cannot breath comfortably |  |  |  |  |
| 1. Cough or snore loudly |  |  |  |  |
| 1. Feel to cold |  |  |  |  |
| 1. Feel to hot |  |  |  |  |
| 1. Have bad dreams |  |  |  |  |
| 1. Have pain |  |  |  |  |
| 1. Other reason(s), please describe, including how often you have had trouble sleeping because of this reason(s) |  |  |  |  |
| 1. During the past 30 days, how would you rate your sleep quality overall? | Very  good (0) | Fairly good (1) | Fairly  bad (2) | Very  bad (3) |
|  |  |  |  |  |
| 1. During the past 30 days, how often have you taken medicine to help you sleep? |  |  |  |  |
| 1. During the past 30 days, how often have you had trouble staying awake while driving, eating meals, or engaging in social activity? |  |  |  |  |
| 1. During the past 30 days, how much of a problem has it been for you to keep up enthusiasm to get things done? |  |  |  |  |

| **Component** | **Item** | **Score** | | | |
| --- | --- | --- | --- | --- | --- |
|  |  | **0** | **1** | **2** | **3** |
| A.subjective sleep quality | #6 score | □Very  good | □Fairly  good | □Farily  good | □Very  bad |
| B.sleep latency | #2 Score + #5a Score | □0 | □1~2 | □3~4 | □5~6 |
| C. sleep duration | #4 Score | □＞7h | □6~7h | □5~6h | □＜5h |
| D.sleep efficiency | (total # of hours asleep)/( total # of hours in bed) | □＞85% | □75~85% | □65~75% | □＜65% |
| E.sleep disturbance | # sum of scores 5b to 5j | □0 | □1~9 | □10~18 | □19~27 |
| F.sleep medication use | #7 Score | □0 | □＜once a week | □Once or twice a week | □≥Three times a week |
| G.daytime dysfunction | #8 score + #9 score | □0 | □1~2 | □3~4 | □5~6 |

**PSQI Score=A+B+C+D+E+F+G**
